# Atlanta Academy of Medicine

**Published:** 1879-04

**Authors:** 


### Reports of Societies.

### ATLANTA ACADEMY OF MEDICINE.

ATLANTA, March 10, 1879.

The Academy was called to order by the Second Vice-President, Dr. A. W. Calhoun.

After transaction of regular business, the following cases were reported:

Dr. J. T. Johnson reported operation for visical calculus.

The patient, a boy aged seventeen, some four years ago, had gotten a bean into his urethra, along which it had travelled into the bladder. Calculus had formed around this as a nucleus, and had reached the size of end of middle finger.

Dr. Salm said: It appears from the last reporter's abstract, that Dr. Wilson apprehends danger from the administration of the sulphate of cinchonidia. He has had a very different experience. While practicing in the country had constant occasion to use the cinchonidia sulph. in substitution for quinia sulph., and found it, when given in somewhat increased dose, equally efficacious. Has given it in ten grain doses, combined with a little Dover's powder, every one to three hours. Thinks it has better effect when given with Dover's powder; commonly gives it in five grain doses.

Dr. Logan has very little experience personally with the cinchonidia, but two or three weeks ago, while on a visit to Florida, had met with a medical gentleman who lived in a very unhealthy and malarial district, and this gentleman informed him that he gave the cinchonidia in same doses as quinia sulph. and with same effect. So much so that he had altogether abandoned the quinia; on the strength of this experience of a trustworthy observer, he has given it in several instances since, but has not had time to see effects. The profession, very generally, Dr. Logan thinks, regards the cinchonida as having less bad after effects than quinia, and the experience of his medical friends bears out the idea.

Dr. Johnson has not used the chinchonidia, but has enquired about it from various sources recently—from men who live in malarial districts. These attach nearly as much value to it as to the quinia when given in slightly increased doses. Has noticed, however, that when they met with very severe cases, fell back on quinia; were fully satisfied with its potency in ordinary cases, but in very difficult ones fell to quina as having no doubts resting over it. Dr. Johnson would not attach any importance to bad effect, noted in Dr. Wilson's case—as same effect sometimes follow use of quinia.

Dr. Logan was very much impressed a short while ago with the action of el. ext. ergot, in menorrhagia; the case was protracted and severe; after various remedies, local, hygeinice, etc., were used, without benefit, gave patient fl. ext. ergot, in drachm doses, three times a day; saw no good results for several days, but persevered when improvement began, and patient soon recovered; remedy kept up for ten days or two weeks; had miscarried about three months ago, and since that time had been constantly suffering from menorrhagia; the miscarriage occurred about the sixth week or the second month, and thinks they occur most often at this time; no special enlargement; has been two months since treatment ceased, and there is no return.

Dr. Westmoreland thinks hemorrhages after abortion, —are caused by relaxation of uterus—a kind of subinvolution; has always used Ergot; the ergot does not act immediately; ergot contracts and gives tone to uterus; knew of case of menorrhagia of years duration that yielded to a month's course of ergot; Dr. Baird asked the gentlemen if they had used ergot for after pains; Dr. Logan has not used it.

Dr. Baird has used it in several cases of after pains and relieved them; in one case after opium had been used ineffectually for two days; accounts for it by ergot, causing continuous tonic contraction; the pains had been caused by relaxation, followed by contraction.

Dr. Johnson asked if no blood had been expelled; Dr. Baird replied in the negative; Dr. Johnson thought that pains were caused by clots of blood in uterus.

Dr. Logan agreed with Dr. Johnson, and also stated, that he had, in several instances, thought the after pains caused by the egot. Dr. Westmoreland said, after pains grow worse the more children the woman has had. After each labor a woman has the uterus is more and more relaxed. Some irritation as blood causes the spasmodic contraction, and the spasmodic contraction causes the after pains. These pains recur every fifteen or twenty minutes. Ergot produces a continuous contraction. When given early in labor, ergot often causes death of child by tonic contraction of uterus, stopping supply of blood going to fœtus. Cannot see how ergot can cause after pains; rather think it will prevent them. Dr. Baird has not seen the remedy in print, and the results were so marked in his cases that he accepted it.

Dr. Westmoreland related two cases, one of femoral and one of umbilical hernia. First case—Femoral hernia in lady residing down on Georgia Railroad. Called to see her on 9th of January, the trouble having existed since the 1st of January. The history was the same as in all strangulated hernia until the day previous to his seeing her, when tumor began to form above Ponpart's ligament. The enlargement was gradual; skin over enlargement inflamed. Examination revealed all the symptoms of strangulated and a femoral hernia. The pulse 130 140 beats in the minute. Patient very much prostrated. Question was whether to operate or let her die without it, but decided to operate. When incision was made through skin and cellular tissue, large quantities of pus and fecal matters were discharged. Continuing incision down to saphenous opening, came upon two knuckles of gut. The strangulation was at the saphenous opening, none at the femoral ring. The knuckles had sloughed off, and was feared the ends would be drawn back into the abdominal cavity and set up fatal inflammation. This was prevented, and the cavity of the abscess well washed out. For first week patient improved very much. On ninth or tenth day took ill and died in a few days. No *post-mortem*. Thinks that one end of the intestine had been drawn into the abdominal cavity.

The second case was one of umbilical hernia, occurring in a very fleshy lady of sixty years. Had reduced this hernia for her eighteen years ago, and she had worn a pad since. This truss did not fit well, and in consequence the intestine had protruded more or less. Had been troubled occasionally with slight inflammation, but it passed over. Strangulation occurred twenty-four hours before operation. Incision was made over tumor, and intestine found to be twisted upon itself so as to remind one of umbilical cord, and four knuckles were formed by this twisting. The intestine had become attached to the peritoneum. Broke up most of them with finger. Found obstruction, but not at umbilical opening. This obstruction was caused by peritoneum and adventitious tissue adherent to the twisted intestine, and, as shown by *post-mortem*, was four or five inches in breadth. Cut the stricture with probe-pointed bistoury on one side and on other, and returned gut, but stricture had not been entirely severed, as shown by *post-mortem*. Attempted to raise the protruding intestine so as to reach the bottom of constriction, but did not accomplish it. The four knuckles were held by four strictures. Patient died in forty-eight hours after operation.

Academy adjourned.

---

ATLANTA, GA., March 24, 1879.

The Academy called to order by the President, Dr. J. S. Todd.

After dispatching the regular order of business, report of cases called up.

Dr. Scott reported case of a gentleman 28 years of age sent to him from Walton county by the attending physician. Some three years ago patient had felt a "boil" in his right ear; picked it and blood was discharged. Again forming, was opened by some physician who used caustic very freely and very bunglingly—so much so that it caused inflammation of all the surrounding parts, and as result of this large cicatrix. Last March ('78) went to another physician, who, finding a polyp had formed, removed it. The polyp formed again, and was again removed in August. Dr. Scott saw the patient last Saturday. The attachment of polyp was at junction of osseous and cartilaginous portions of canal. The tumor was about the size of a pigeon egg and looked like fungus; was very vascular and had the appearance of malignancy; had sloughed several times, which process was followed by profuse hemorrhage.

Diagnosis—malignant polyp.—Applied Wilde's snare, but the instrument breaking he used a pair of scissors for its removal. Profuse hemorrhage followed, but checked with Fr. chloride. Then with a curette he scraped the part where the tumor was attached; again checked the hemorrhage with same hæmostatic, and next applied a caustic composed of equal parts of chloride and oxide of zinc, with a little morphine added. Would recommend this caustic, as it seems to possess the faculty of selecting between the healthy and diseased tissue.

Dr. Salm asked whether cutting or twisting was the usual method of removing these polypi.

Dr. S. replied that twisting was the usual practice, but that his snare had broken. Had recommended the attending physician to use argenti nitras as an after treatment. In answer to question said that if pinna were pulled back, could hear very well; otherwise tumor obstructed the hearing. Since operation, hearing perfectly good. Periodically the tumor became painful, for the relief of which the patient simply pricked it, thereby allowing some of the blood to escape.

Dr. Wilson stated that the peculiar effects caused by the administration of cinchonidia sulph., in the case related by himself, did not seem very unusual to some of the other gentlemen; that they had known like results to follow the use of quinia. Accordingly asked them to relate the cases.

Dr. J. T. Johnson had noticed as effects of quinia eruptions, tinitus, aurium, and headache, and had read of general nervous symptoms very similar to those given in Dr. Wilson's case, where there was loss of speech, tremor, coldness of extremities, etc.

Dr. Wilson had heard of three other instances since that time where similar effects followed the use of cinchonidia.

Dr. Salm said that quinia had caused in so many cases temporary deafness and headache that he had resorted to the cinchonidia, and with less bad effects.

The President asked Dr. Salm if he had noticed dilatation of pupil after administration of cinchonidia.

Dr. S. said he had not.

Dr. Calhoun related a case of a woman 34 years of age who, when 5 or 6 years old, had been operated on for strabismus by some traveling medical man. The ball after the operation was drawn to the outer side more than it had been to the inner—so much so that the pupil was partly obscured by the outer canthus. Could even feel the optic nerve, so much had the eye been everted. Dr. C. was very doubtful as to success, but chloroformed her and cut through the conjunctiva to ascertain if the muscle, the internal rectus, still was present. Did not find it, and occurred to him that it might have receded and not attached itself to ball after having been severed in the original operation. This proved to be the case, and accounted for the fact that the eye had been drawn so far to the other side. With a tenaculum he caught the end of the long unused muscle, dissected it up, and passed a couple of sutures through its end to draw it forward; then divided the external rectus and atttached by sutures the internal rectus and conjunctiva. The eye was brought almost into its correct position by this procedure, and has done well since. The internal rectus attached itself, where it was held by the sutures. The external muscle attached itself to the ball after receding. The eyesight, much to his surprise, was not lost, but was not perfect by any means. Thought the trouble originally was that the muscle had been cut too far back into the belly. Since recovery, has perfect use of eye as regards movements.

Dr. Mack thought it singular that the muscle had not atrophied after being idle for twenty-eight years.

Academy adjourned.

ATLANTA, GA., March 31, 1879.

Academy called to order by President Todd.

After the reading of an Essay by Dr. Scott and a few remarks thereon by the members present, Dr. Wilson made the following remarks, to-wit:

Some months previously there had been sent to him for trial a package of sulphate of cinchonidia, and he had used it in his own family and servants with satisfactory results. Two weeks ago, however, he administered it to a lady patient at Kirkwood, directing it to be given between four and five grains in capsules every two hours. She took only one capsule, and in fifteen minutes there ensued a tremor of body and extremities, sharp and darting pains, especially over region of heart. Patient was very pale, and for an hour there was almost complete loss of power of speech. So distressing were her symptoms that her husband and their neighbors thought she was poisoned.

Dr. W. saw her again late in the evening, and there was still a disposition to paroxysms of pain, though alternating with intervals of complete rest. He gave her pretty strong doses of McMunn's elixir opium.

Dr. Wilson said the cinchonidia was undoubtedly a pure article; was impressed with the idea that the symptoms were due to cinchonidia.

Dr. Cotelyon has administered this remedy several times, and without any unpleasant effects. It failed to break up fever in his own children, and he had resorted to quinine.

Dr. Wilson stated that several cases similar to the one reported by him had occurred in the practice of Dr. Raines from administration of cinchonidia.

Dr. Cotelyon said that quinine was sometimes followed by like unpleasant symptoms. One patient became so wild after taking it that he was accused of having given morphine. In another patient only an exceedingly minute dose could be borne.

The President likewise spoke of the peculiar effects of quinine on some constitutions. Used in larger doses, it makes the head giddy, and, in common with cinchonidia and quinidia, dilates the pupil, unless a little morphine is added.

Academy adjourned.

